# HIV Nef-mediated Ubiquitination of BCL2: Implications in Autophagy and Apoptosis

**DOI:** 10.3389/fimmu.2021.682624

**Published:** 2021-05-06

**Authors:** Sergio Castro-Gonzalez, Sydney Simpson, Yuhang Shi, Yuexuan Chen, Jared Benjamin, Ruth Serra-Moreno

**Affiliations:** Microbiology and Immunology, University of Rochester Medical Center, Rochester, NY, United States

**Keywords:** autophagy, apoptosis, HIV, Nef, BCL2, BECN1, PRKN

## Abstract

Ubiquitination is a process that acts upon every step of the HIV replication cycle. The activity, subcellular localization, and stability of HIV dependency factors as well as negative modulators can be affected by ubiquitination. These modifications consequently have an impact on the progression and outcome of infection. Additionally, recent findings suggest new roles for ubiquitination in the interplay between HIV and the cellular environment, specifically in the interactions between HIV, autophagy and apoptosis. On one hand, autophagy is a defense mechanism against HIV that promotes the degradation of the viral protein Gag, likely through ubiquitination. Gag is an essential structural protein that drives virion assembly and release. Interestingly, the ubiquitination of Gag is vital for HIV replication. Hence, this post-translational modification in Gag represents a double-edged sword: necessary for virion biogenesis, but potentially detrimental under conditions of autophagy activation. On the other hand, HIV uses Nef to circumvent autophagy-mediated restriction by promoting the ubiquitination of the autophagy inhibitor BCL2 through Parkin/PRKN. Although the Nef-promoted ubiquitination of BCL2 occurs in both the endoplasmic reticulum (ER) and mitochondria, only ER-associated ubiquitinated BCL2 arrests the progression of autophagy. Importantly, both mitochondrial BCL2 and PRKN are tightly connected to mitochondrial function and apoptosis. Hence, by enhancing the PRKN-mediated ubiquitination of BCL2 at the mitochondria, HIV might promote apoptosis. Moreover, this effect of Nef might account for HIV-associated disorders. In this article, we outline our current knowledge and provide perspectives of how ubiquitination impacts the molecular interactions between HIV, autophagy and apoptosis.

## Ubiquitination in the Mutual Antagonism Between HIV and Autophagy

Ubiquitination post-translational modifications are used for multiple purposes including signaling transduction, enhancing protein function, driving protein subcellular localization and targeting proteins for degradation (1–3). One of these degradation pathways is autophagy, which targets components in the cytosol, including subcellular organelles and microbial pathogens, for lysosomal degradation. Whereas Lys-48 polyubiquitination commonly directs substrates to the proteasome (4–7), autophagy cargos are usually tagged with Lys-63 ubiquitin chains (4, 7, 8). Specifically, Lys-63 polyubiquitinated molecules are recognized by the ubiquitin binding domain (UBD) of different specialized autophagic receptors, including SQSTM1/p62. These receptors can simultaneously bind to ubiquitinated cargo and autophagosomal markers (i.e., LC3), allowing for the encapsulation of substrates into elongating autophagosomes (9–11).

**Figure 1:**
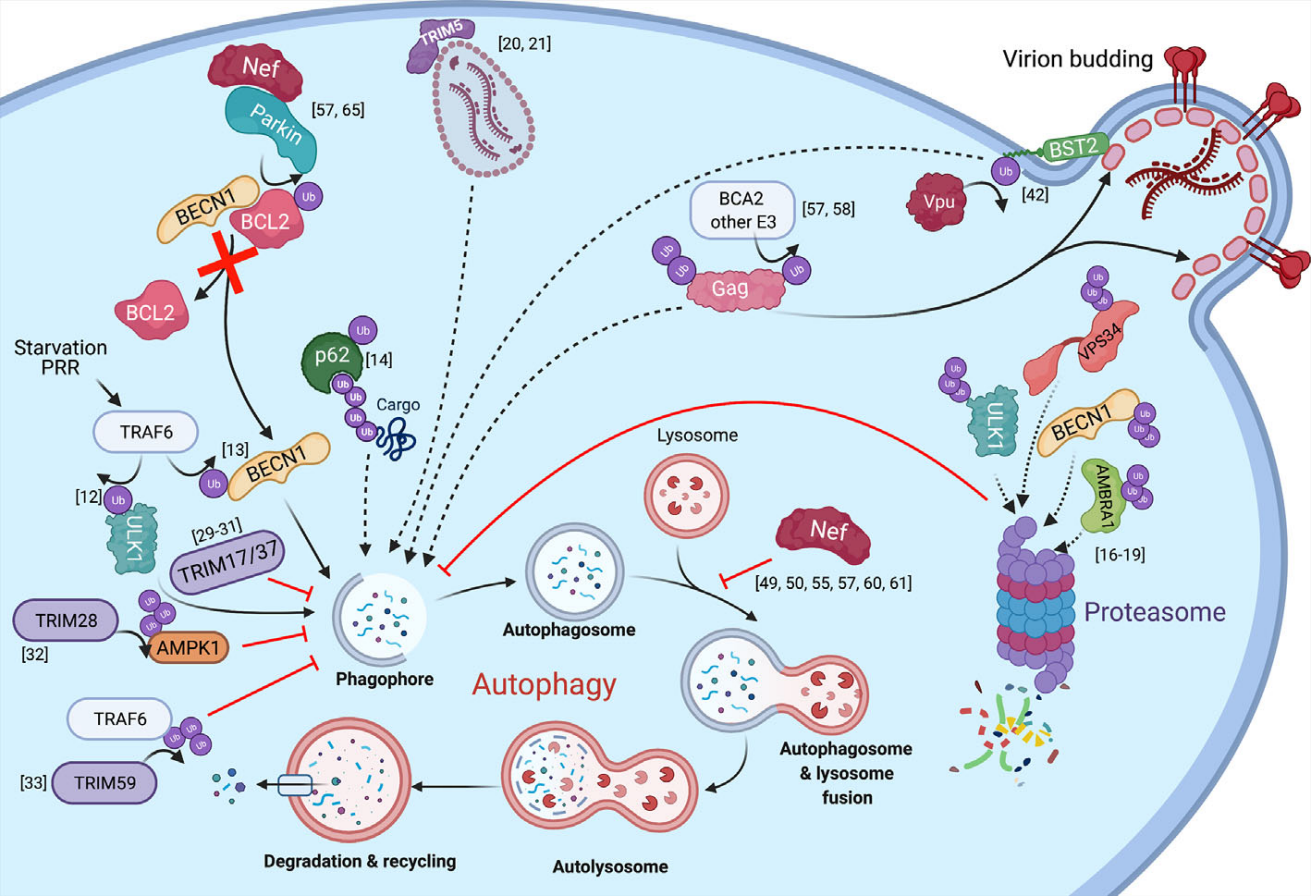
The intersections between HIV infection and autophagy mediated by ubiquitination. The autophagy pathway and known interactions with HIV proteins are shown with regards to ubiquitination. Solid arrows represent the transfer of a ubiquitin group, direct contribution to the autophagy pathway, or show the steps of the autophagy cycle. Dotted lines represent substrates targeted for elimination. Red lines or arrows represent autophagy inhibition at corresponding steps in the pathway.

Autophagy itself is highly regulated, in part through ubiquitination, as well as other post-translational modifications (1). For instance, ULK1, a serine/threonine kinase responsible for inducing autophagy under conditions of amino acid withdrawal, is ubiquitinated by the E3 ubiquitin ligase TRAF6, which enhances ULK1’s function and stability (12). Moreover, TRAF6 ubiquitinates Beclin1/BECN1, which promotes autophagy induced by Toll-like receptor 4 signaling (13). p62 activity can similarly be regulated through E2-supported ubiquitination by UB2D2/UB2D3, which allows for this receptor to recognize cargo (14) (**Figure 1**). In addition to triggering autophagy, ubiquitination can also down-regulate this pathway by targeting components of this cascade for degradation (15). Particularly, the ubiquitination of (i) ULK1 and VPS34 by the E3 ligase Cul3-KLHL20, (ii) BECN1 by RNF216, and (iii) AMBRA1 by Cullin-4, promotes the proteasomal degradation of these autophagy regulators and, thus, reduces autophagy flux (16–19) (**Figure 1**). Additionally, many members of the tripartite motif (TRIM) family of E3 ligases regulate autophagy, although their mode of action does not always involve ubiquitination. Among the TRIM proteins that positively modulate autophagy we find TRIM5α, TRIM6, TRIM16, TRIM17, TRIM20, TRIM21, TRIM22, TRIM23, TRIM49 and TRIM50. These molecules trigger autophagy in response to stimuli such as viral infections, cell damage, IFNγ stimulation and pattern recognition receptor (PRR) engagement (20–28). Examples of TRIM members that down-regulate autophagy are TRIM17, TRIM28, TRIM37 and TRIM59. Despite its ability to promote autophagy of midbodies, TRIM17 can also hinder autophagy by stabilizing the autophagy inhibitor MCL1. In an analogous manner, TRIM37 increases the stability of mTOR, which naturally keeps autophagy off (29–31). By contrast, rather than enhancing the activity of autophagy inhibitors, TRIM28 and TRIM59 prevent autophagy through the ubiquitination and subsequent degradation of the positive regulators AMPK1α and TRAF6, respectively (32, 33) (**Figure 1**). Although not through ubiquitination, LC3 – a key player in autophagy initiation, progression and execution – is regulated through a ubiquitin-like process. Particularly, LC3 becomes lipidated and this post-translational modification requires the concerted action of enzymes that mimic the function of the E1, E2 and E3 enzymes involved in ubiquitination, and they are often referred to as E3-like ligases (34–36).

Ubiquitination also plays a role in viral infections, including HIV. On one hand, the HIV accessory proteins Vif, Vpr, Vpx and Vpu usurp ubiquitination signaling to target host factors – which would otherwise limit virion production – for proteasomal or lysosomal degradation (37–41), and also for autophagy-mediated clearance (42). For instance, Vpu has been reported to re-route the restriction factor Tetherin/BST2 to autophagosomes in a non-canonical manner (**Figure 1**). On the other hand, ubiquitination can cause the degradation of HIV proteins through the proteasomal, lysosomal and/or autophagy pathways. For example, the ubiquitination of the HIV core by TRIM5α promotes the proteasomal degradation of the capsid and premature termination of reverse transcription. In addition to this well-known activity, TRIM5α also triggers autophagy in response to HIV infection and leads to the autophagic degradation of the HIV core (20, 21) (**Figure 1**). Another example of ubiquitin-dependent degradation is that of the HIV protein Tat, which is responsible for switching from a state of viral latency to one of productive replication. Tat is ubiquitinated by the E3 ligase CHIP, which consequently causes its proteasomal degradation, and thus, negatively impacts viral transcription (43). However, Tat is also susceptible to ubiquitination in a non-degradative manner by the E3 ligase PJA2. Specifically, PJA2 adds atypical ubiquitin chains to Tat that increase its transactivating potential (44). Besides being degraded at the proteasome, Tat has also been found as an autophagy target in CD4^+^ T cells, although its ubiquitination is not required to route it to autophagosomes (45). Conversely, Tat has been reported to down-regulate autophagy in neurons (46). Similar to Tat, Vif is also directed for autophagy-mediated clearance in a ubiquitin-independent manner through an association with the HDAC6 deacetylase (47). Paradoxically, Vif has been reported to inhibit canonical autophagy by associating with LC3 and preventing its incorporation into autophagosomal structures. Although the underlying mechanism by which Vif achieves this is not well understood, it does not seem to rely on LC3 ubiquitination or degradation (48).

The dichotomy of Tat and Vif in their relationship with the autophagy machinery underscores that the autophagy-HIV interactions are complex. Actually, conflicting reports exist regarding this interplay, with studies supporting that autophagy enhances or inhibits the progression of infection, depending on cell type (20, 45, 49–56). However, our group recently found that the HIV Gag polyprotein, the immature precursor of several structural proteins required for virion maturation, is degraded through autophagy regardless of cell type (57). Since Gag is susceptible to ubiquitination, which is part of its role in facilitating virion budding, it is likely that the targeting of Gag to autophagosomal membranes requires ubiquitination as well. In fact, our previous work on breast cancer-associated gene 2 (BCA2), a RING finger E3 ubiquitin ligase, showed that this protein promotes the ubiquitination and subsequent lysosomal degradation of HIV Gag (58). Hence, Gag ubiquitination could similarly tag this protein for autophagy-mediated clearance (**Figure 1**). Despite the antiviral potential of autophagy against HIV, our work also revealed that the virus has evolved mechanisms to counteract the autophagy-mediated destruction of viral elements needed for replication, including Gag. In particular, the HIV protein Nef counteracts the antiviral effects of autophagy. Importantly, while Nef is not required for replication *in vitro*, it enhances infection and contributes to pathogenesis *in vivo* by affording immune evasion through multiple mechanisms, including MHC-I down-regulation and counteraction of the restriction factors SERINC3 and SERINC5 [reviewed in (59)]. Besides these roles, Nef was also known to prevent viral degradation caused by autophagy through (i) a physical association with BECN1, inhibiting in turn autophagolysosomal biogenesis, and (ii) by promoting the cytosolic sequestration of the transcription factor TFEB, a master regulator of autophagy genes (49, 50, 55, 60, 61). In both models, the ultimate outcome is a defect in the fusion between autophagosomes and lysosomes. In support of these findings, we also found that Nef blocks autophagy maturation. However, we uncovered an additional mechanism by which Nef blocks autophagy at early stages of the cascade, and that requires a physical association between Nef and the E3 ubiquitin ligase Parkin/PRKN. Specifically, Nef recruits PRKN to promote the mono-ubiquitination of BCL2, an autophagy inhibitor (**Figure 1**) (57). Under normal conditions, BCL2 interacts with the autophagy initiator BECN1 to prevent autophagosome formation. However, under conditions of stress, BECN1 dissociates from BCL2 to initiate autophagosome biogenesis (62–64). Remarkably, the sequestration of BECN1 by BCL2 is enhanced if BCL2 is mono-ubiquitinated (65). In fact, we found that the Nef-mediated recruitment of PRKN not only increases BCL2 ubiquitination but also BECN1-BCL2 association. As a consequence of this, autophagosome formation is severely inhibited, while Gag levels and virion production are restored (**Figure 1**) (57). Based on these observations, we conclude that, besides its already described roles in autophagy maturation, Nef circumvents the autophagy block by intersecting with an early event in the autophagy cascade.

## Effects of the Nef-Promoted Ubiquitination of BCL2 on Autophagy and Apoptosis

**Figure 2:**
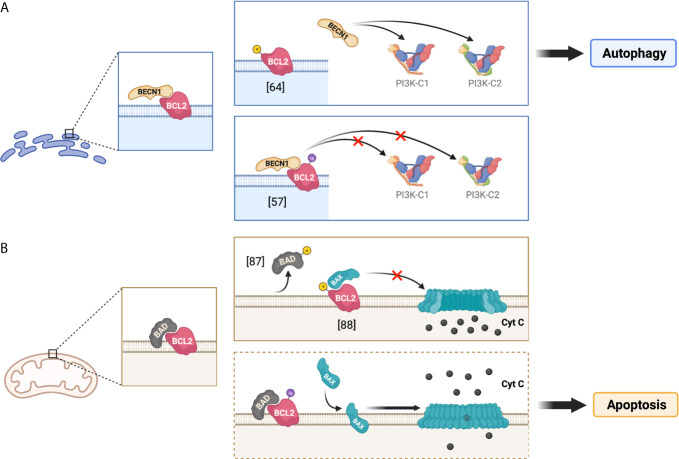
Implications of the phosphorylation and ubiquitination of BCL2 at the ER and mitochondria. The figure illustrates the regulation of BCL2 functions through its post-translational modifications at different subcellular localizations, depicting the potential effect of BCL2 ubiquitination on autophagy and apoptosis. **(A)** presents the roles of BCL2 in the endoplasmic reticulum (ER), whereas **(B)** depicts the roles of BCL2 at the mitochondria. The dotted frame in **(B)** represents putative roles.

The increased levels of ubiquitinated BCL2 promoted by Nef may have further implications for infected cells. BCL2 is found in multiple subcellular compartments, including the endoplasmic reticulum (ER) and the mitochondria (66–68). ER-associated BCL2 is mainly involved in the regulation of autophagy through its interaction with BECN1, as stated above. Specifically, BCL2 binds to the BCL2 homology domain 3 (BH-3 domain) of BECN1 and sequesters this molecule, which in turn impairs autophagy at both initiation and maturation stages. Autophagy initiation and maturation are dependent on the respective formation of the protein complexes PI3K-C1 and PI3K-C2 (class III PI 3-kinase complex 1 and complex 2). The core structure of PI3K-C1 consists of VPS34, BECN1, VPS15, and ATG14. PI3K-C2 core structure differs from C1 by the absence of ATG14 and the addition of UVRAG. The presence of ATG14 or UVRAG targets each complex towards phagophore membranes or autophagosomal membranes, which helps modulate autophagy initiation and maturation, respectively (69–71). Besides its role in sequestering BECN1, BCL2 may additionally intersect with C1/C2 and inhibit VPS34 kinase activity by blocking BECN1 interactions with ATG14 and UVRAG in their respective complexes (57, 69, 72, 73). The resulting effect of BCL2 binding to BECN1 is an overall antagonization of autophagy. Although this phenomenon has been described in more detail for C1 formation, the sequestration of free BECN1 might also impact the biogenesis of C2 and, thus, autophagy maturation (**Figure 2A**). Therefore, a major point of regulation in autophagy is the promotion and reduction of BECN1-BCL2 complexes.

Besides its ability to associate with the ER, BCL2 and other members of the BCL2 family can also be found at the mitochondria, where they regulate apoptosis by controlling the permeabilization of the outer mitochondrial membrane. Apoptosis is an essential process that removes damaged or infected cells in an orderly fashion. Under conditions of extreme stress or damage, pro-apoptotic effector proteins, such as BCL2-associated X protein (BAX) or BCL2 antagonist/killer-1 (BAK), homo-oligomerize to form pores on the outer mitochondrial membrane (74–77). The formation of these pores allows for cytochrome C to escape from the mitochondrial lumen. The release of cytochrome C to the cytosol enables its binding to the apoptotic protease activating factor (Apaf-1) and consequently triggers multiple caspase cascades that lead to this controlled death fate (77–79). Conversely, under healthy conditions, BCL2 pro-survival proteins directly bind to the BH3 domain in BAX to prevent pore formation on the outer mitochondrial membrane, which in turn prevents apoptosis (80, 81). Due to the importance of maintaining a healthy cell survival/cell death equilibrium, there are other relevant cellular factors involved in this apoptotic regulation. In particular, the BH3 domain of BCL2-associated agonist of cell death (BAD) is able to directly bind to mitochondria-associated BCL2 to hamper its anti-apoptotic function (82–84) (**Figure 2B**). Therefore, the BCL2-mediated inactivation of BAX, as well as its own regulation *via* BAD interaction, are crucial to control mitochondrial membrane integrity and consequently cell survival.

Whereas its subcellular localization dictates which cellular pathway will be susceptible for BCL2-mediated inhibition (autophagy *vs.* apoptosis), post-translational modifications of BCL2 will regulate the degree of restriction exerted over these two pathways. From this perspective, BCL2 is susceptible to become phosphorylated at Ser70 by the JNK1 kinase (85–87). When this phosphorylation occurs at the ER, it causes a reduction in the association between BCL2 and BECN1 and thus, it facilitates autophagy activation and progression by increasing the availability of BECN1 (62, 64, 85) (**Figure 2A**; top). Alternatively, JNK1 as well as other kinases have been reported to mediate the phosphorylation of both BCL2 and its inhibitor BAD at the outer mitochondrial membrane (86, 88). This phosphorylation can promote the dissociation of BCL2 from BAD, which enables BCL2 anti-apoptotic function by increasing the ratio BCL2 to BAX (86, 88) (**Figure 2B**; top).

As noted above, in addition to phosphorylation, BCL2 is mono-ubiquitinated by PRKN. Of note, PRKN primarily localizes at the mitochondria where it helps regulate mitochondrial quality control, namely through mitophagy – the autophagy of mitochondria – when these organelles are impaired (89–91). The Nef-promoted mono-ubiquitination of BCL2 at the ER increases its stability and enhances its association with BECN1 to further restrict the early stages and progression of the autophagy pathway (57, 65) (**Figure 2A**; bottom). Besides facilitating BCL2 mono-ubiquitination at the ER, HIV Nef also increases mono-ubiquitination and stability of the BCL2 isoform in the mitochondria, although it does not cause an enhancement in its association with BECN1 (57). However, the potential impacts of mitochondrial BCL2 mono-ubiquitination on mitophagy and apoptosis are yet to be determined. One could speculate in this matter that, analogously to what applies for the ER in terms of BCL2-BECN1 interactions, the mono-ubiquitination of BCL2 at the mitochondria might promote BCL2-BAD association and consequently, facilitate cell death through apoptosis (**Figure 2B**; bottom).

## Discussion and Concluding Remarks

The effects of the Nef-dependent mono-ubiquitination of BCL2 could have strong implications in our understanding of the role of Nef in the development of pathologies associated with HIV^+^ individuals. First of all, the fact that not only Nef impacts autophagy restriction but also might facilitate apoptotic events through the PRKN-dependent mono-ubiquitination of BCL2 could provide mechanistic support to previous studies that connect Nef expression and apoptosis in different cell types, such as cardiomyocytes, brain endothelial cells or CD4^+^ T cells (92–94). Importantly, this Nef-mediated apoptosis may be responsible, at least in part, for the depletion of infected as well as bystander CD4^+^ T cells, which is the main cause of the immunodeficiency observed in infected individuals. Additionally, the effect of the Nef-enhanced ubiquitination of BCL2 on autophagy could also account for the development of other HIV-associated pathologies, including HIV-associated neurological disorders (HAND) or HIV-associated pulmonary hypertension (HIV-PH) (95–97). These conditions are not only relevant because of their undesired symptomatology and prognosis, but also because they are usually associated with persons living with HIV, even those controlling the infection due to their adherence to the antiretroviral regimens. Whereas Nef expression has been linked to the development of these pathologies, the underlying mechanisms still remain unknown. Remarkably, several studies have demonstrated that autophagic dysfunction is directly associated with pulmonary hypertension as well as numerous neurological and cognitive conditions, including HAND (96, 98, 99). In fact, autophagy dysregulation by HIV Tat has been linked with neuronal degeneration (46), so the effect of Nef on BCL2 ubiquitination and its role in autophagy might exacerbate neuronal damage. In addition, malfunction of the ubiquitin ligase PRKN seems to be associated with neurological disorders (100, 101). Therefore, Nef’s capacity to intersect with autophagy through the recruitment of PRKN to drive the mono-ubiquitination of BCL2 might be a key driver in the development of these HIV-associated conditions.

Ubiquitin has a relevant position in the arms-race between HIV and the host cell environment. Despite being required for viral particle assembly, the ubiquitination of HIV Gag might be tagging this protein for the so-called ‘kiss of death’ fate. Conversely, HIV has evolved a strategy to hijack the cellular ubiquitin machinery in order to overcome this hurdle. This mechanism involves the mono-ubiquitination of BCL2 that, in addition to preventing the degradation of Gag, might also be lifting the endogenous control over apoptosis. This in turn could have a wide range of implications for the pathogenesis and prognosis of HIV^+^ individuals. Therefore, further elucidation of the role of ubiquitin within the interplay between HIV and the host could be promising for the identification of new therapeutic targets against this virus.

## Author Contributions

SC-G, SS, and RS-M outlined the perspectives paper. SC-G, SS, YS, YC, JB, and RS-M researched specific sections of the perspectives article and provided the literature referenced here. SC-G wrote the abstract. SC-G and SS designed the figures. SC-G, SS and RS-M wrote the manuscript. All authors contributed to the article and approved the submitted version.

## Funding

We thank the University of Rochester Medical Center (URMC) for supporting our work. SC-G is supported by a NIH HARC collaboration grant (P50AI150476; subcontract award 12310sc; Serra-Moreno PI), YS is supported by NIH R21AI138589 (Serra-Moreno, PI), YC is supported by NSF RAPID MCB-2032518 (Serra-Moreno, PI). JB and SS are supported by RS-M startup funds. RS-M is supported by URMC, P50AI150476, R21AI138589, and MCB-2032518.

## Conflict of Interest

The authors declare that the research was conducted in the absence of any commercial or financial relationships that could be construed as a potential conflict of interest.

The handling editor has declared a past collaboration with one of the authors [RS-M].
